# Outcomes Following Salvage Radiation and Systemic Therapy for Isolated Locoregional Recurrence of Breast Cancer after Mastectomy: Impact of Constructed Biologic Subtype

**DOI:** 10.1155/2018/4736263

**Published:** 2018-09-12

**Authors:** Xiaofang Wang, Jinli Ma, Xin Mei, Zhaozhi Yang, Xiaoli Yu, Xiaomao Guo, Zhen Zhang, Zhimin Shao

**Affiliations:** ^1^Department of Radiation Oncology, Fudan University Shanghai Cancer Center, Shanghai, China; ^2^Department of Oncology, Shanghai Medical College, Fudan University, Shanghai, China; ^3^Department of Breast Surgery, Fudan University Shanghai Cancer Center, Shanghai, China

## Abstract

**Purpose:**

This study examines factors associated with outcomes following salvage radiation and systemic therapy for breast cancer patients who developed isolated locoregional recurrence (ILRR) after mastectomy alone, while focusing on the prognostic significance of constructed biologic subtype in this setting.

**Methods and Materials:**

269 postmastectomy patients in total treated for ILRR were included. Cumulative incidence of locoregional control (LRC), distant metastasis (DM)-free survival (DMFS), disease-free survival (DFS), and overall survival (OS) were calculated using Kaplan-Meier method. For statistical analysis, biologic subtypes were constructed from hormonal receptors (Rec) and HER2, consisting of Rec+/HER2-, Rec+/HER2+, Rec-/HER2+, and Rec-/HER2-. The association of clinic-pathological and treatment-related parameters with outcomes was evaluated using a Cox regression model.

**Results:**

At a median follow-up of 65 months, 56 (20.8%) patients failed to secure LRC after radiotherapy, and 165 patients (61.3%) developed DM. Overall, the actuarial 5-year LRC, DMFS, DFS, and OS rate was 77.3%, 45.6%, 43.9%, and 66.8%, respectively. Multivariate analysis revealed that constructed biologic subtype represented the most significant prognostic factor for any outcome. Compared to patients with Rec+/HER2- disease, those with Rec-/HER2- had significantly poorer 5-year LRC (84.2% versus 58.3%, HR = 4.36, P < 0.001) and worse survivals including 5-year DMFS (63.0% versus 15.8%, HR = 4.28, P < 0.001), DFS (59.7% versus 13.6%, HR=3.92, P < 0.001), and OS (87.8% versus 22.3%, HR = 8.55, P < 0.001). Other factors associated with reduced LRC were no radical surgery and involved field irradiation alone, whereas factors associated with poor survivals included positive nodes at primary diagnosis and regional recurrence.

**Conclusions:**

Constructed biologic subtypes remained to be predictive of both disease control and survivals after salvage radiation for postmastectomy ILRR. Notably, Rec-/HER2- patients were demonstrated to be at high risk of locoregional failure and subsequent DM and tended to have worse survivals despite salvage therapies.

## 1. Introduction

Following mastectomy for operable breast cancer, approximately 5-20 percent of patients will develop a locoregional recurrence (LRR) with or without concomitant distant metastasis (DM) within 10 years [1, 2]. Although multimodality therapy including surgery, radiation, and systemic therapy has the potential to provide long-term disease control for some patients with isolated LRR (ILRR), i.e., LRR without concomitant DM, a substantial proportion of patients develop a subsequent DM with or without a second LRR [3–5]. Previous studies have shown that the outcomes following locoregional and systemic therapies for ILRR after mastectomy might be affected by multiple clinic-pathological factors, such as nodal status at primary diagnosis, hormone receptor status, disease-free interval, and site of ILRR [2, 3, 6–9]. However, the prognostic factors were inconsistent among these studies due to the existing disparities in patient subgroups, treatment details, time period studied, etc. Recent studies have stratified breast cancers into four subtype-approximations defined by receptor expression. These biologic subtypes are increasingly recognized as predictors of disease control after initial treatment [10–13]; however, the implications of biologic subtype in the prognosis following salvage radiation for postmastectomy ILRR are less well studied.

This study was to assess the clinical outcomes following salvage radiation for a series of breast cancer patients who developed ILRR as a first event after initial treatment with mastectomy and adjuvant systemic therapies but without postmastectomy radiotherapy (PMRT) in the contemporary era, while focusing on the prognostic significance of constructed biologic subtype in this setting.

## 2. Materials and Methods

A total of 269 postmastectomy breast cancer patients who had not received any adjuvant radiotherapy and who were treated for ILRR were selected from the database during the period between 2005 and 2014 at our institute. The ILRR is defined as a recurrence within the ipsilateral chest wall (CW) and/or regional nodes (i.e., ipsilateral axilla, supra-/infraclavicular region (SCV/ICV), or internal mammary nodes (IMN)), without concomitant visceral or bony distant metastasis (DM) within 4 months of LRR. All metastases were ruled out by thorough restaging evaluation, e.g., brain computed tomography (CT) or magnetic resonance imaging (MR), chest CT, abdomen ultrasound (US) or MR, and emission computed tomography (ECT). Patients who received previous radiation to the CW and/or regional nodes or had pathology other than breast cancer, with ≥ 4 positive nodes, or with unknown receptor status were excluded from this study. The complete medical records of eligible patients were reviewed by health information management professionals trained in data extraction, and data were collected to determine clinical, pathologic, treatment, and outcome variables. To ensure the accuracy of pathological diagnosis, all primary pathology data were centrally reviewed.

### 2.1. Patient Characteristics

Patient characteristics at the primary diagnosis are shown in Table 1. The initial surgical treatment was modified radical mastectomy (MRM) in 228 patients (84.8%), while 41 patients (15.2%) underwent radical mastectomy (RM). Most patients had a primary tumor (T) classification of pT1-2 (n = 244; 90.7%), and most tumors were moderately to poorly differentiated (n = 251; 93.3%). The median number of nodes examined was 13 (range, 5 - 31), and the distribution of the nodal stages was pN0 and pN1 in 53.5% (n = 144) and 46.5% (n = 125) of patients, respectively.

Basically, biologic subtypes were constructed according to the status of hormonal receptors (Rec) and human epidermal receptor 2 (HER2) from primary tumor specimens. To minimize the effects of status changes, biologic subtypes would be constructed based on Rec and HER2 status from recurrent tumor specimens in patients with surgical biopsy done at time of recurrence. Resultantly, the biologic subtypes consisted of Rec+/HER2- in 58.7% (n = 158), Rec+/HER2+ in 9.7% (n = 26), Rec-/HER2+ in 8.9% (n = 24), and Rec-/HER2- (triple negative) in 22.7% (n = 61) of patients, respectively. Rec+ was defined as ER+ and/or PR+, and Rec- as both ER- and PR-. ER and PR status were evaluated by immunohistochemistry (IHC) staining and were considered positive if IHC staining ≥10% of tumor tissue. HER2 status was determined by IHC staining. Tumors were considered HER2 positive if they scored 3+, on IHC, indeterminate if 2+, and negative if 1+ or 0. When IHC was indeterminate, tumors were considered HER2 positive with amplification (ratio ≥2.0) by fluorescence in situ hybridization (FISH) analysis.

The vast majority of patients had received some kind of adjuvant systemic therapy for their primary diagnosis of breast cancer, consisting of chemotherapy alone in 38.7% (n = 104), endocrine therapy alone in 4.1% (n = 11), and a combination of the two in 54.6% (n = 147) of patients. And very few patients (n = 4) received anti-HER2 therapy, which is largely attributed to unavailability of anti-HER2 medications at the time of mastectomy, or family financial difficulties.

Patient characteristics at diagnosis of ILRR are listed in Table 2. All LRRs were detected by imaging studies and/or physical examinations. Of these, 94.4% (n = 254) were pathologically confirmed from surgical specimens (n = 87) or fine needle aspirations (FNA) (n = 167); and the others were hard to be biopsied and diagnosed clinically based on radiological findings, consisting of 11 positron emission tomography (PET)-CT scans and 4 contrast-enhanced CT scans. The median interval to ILRR from primary diagnosis was 34.2 months. Among all ILRRs, 90 (33.5%) were isolated to CW, 141 (52.4%) were isolated to regional nodes, and 38 (14.1%) occurred in both CW and regional nodes. As an integral part of multimodal treatment, 241 patients (89.6%) received comprehensive locoregional irradiation, and 28 (10.4%) received involved-field irradiation of either CW alone or regional nodes alone. Radiation doses varied from 46 in 23 fractions to 66 Gy in 33 fractions, with the most frequently used dose being at 60 Gy. Before radiotherapy, 80 patients underwent surgical excisions. Of them, 60 were rendered free from gross disease by radical surgery (8 axillary clearance, 38 extended resection of CW lesion, 9 axillary clearance and extended resection of CW lesion, 3 IMN dissection and extended resection of CW lesion, and 1 SCV clearance), and 20 had clinically apparent residual disease following palliative surgery. The majority (95.2%) were given some kind of systemic therapy, including chemotherapy in 202 patients (75.1%), endocrine therapy in 184 patients (69.8%), and anti-HER2 therapy in 24 patients (8.9%).

### 2.2. Statistical Analysis

Follow-up data available as of Dec 31, 2017, were analyzed. The locoregional control (LRC) was defined as freedom from clinical or radiographic evidence of locoregional failure (LRF) within the ipsilateral CW and/or regional draining lymphatics after treatment with radiation for initial ILRR. The distant metastasis-free survival (DMFS) was measured from the date of diagnosis of ILRR to the date of DM. The disease-free survival (DFS) was measured from the date of diagnosis of ILRR to the date of second ipsilateral LRR, DM, or death attributable to breast cancer and/or second primary nonbreast cancer. The OS was defined as the time from the diagnosis of ILRR to death attributable to breast cancer, cause other than breast cancer, or unknown cause at the last follow-up date. Statistics were performed using SPSS software (v18.0). The probabilities of LRC, DMFS, DFS, and OS were calculated using the Kaplan-Meier product-limit method, and compared between groups using the log-rank test. The influence of primary tumor characteristics, recurrence patterns, and treatment-related factors after ILRR on LRC, DFS, and OS were tested by univariate and multivariate analysis using forward step-wise Cox regression method. All p values were two-sided and if less than 0.05 were deemed significant.

## 3. Results

### 3.1. Locoregional Control and Distant Metastasis

At a median follow-up time of 65 months from the diagnosis of recurrence, 56 patients (20.8%) failed to secure LRC. Of these, 53 patients (19.7%) subsequently developed a second LRR, and 3 patients (1.1%) had locoregional progression after salvage RT. As shown in Table 3, the most common site of LRF was CW, followed by regional nodes, basically the SCV/ICV and IMN; and 8 patients had multiple sites of recurrence. From another aspect, the LRFs located in the radiation field were as common as those occurring out of field. However, patients treated with involved fields underwent more “out of field” failures than did those irradiated with comprehensive locoregional fields (in field/out of field/both: 1/9/3 versus 22/10/8, *χ*^2^ = 10.5, P = 0.005).

**Figure 1:**
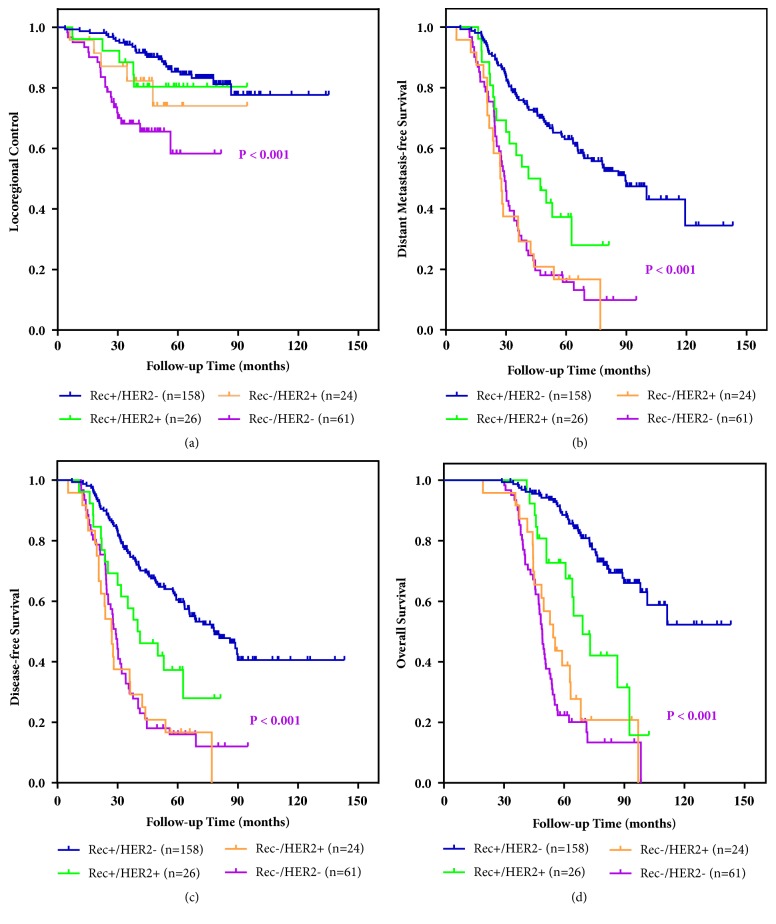
(a) Kaplan-Meier locoregional control (LRC) following salvage radiotherapy for postmastectomy ILRR as a function of constructed breast cancer biologic subtypes; (b) distant metastasis-free survival (DMFS); (c) disease-free survival (DFS); and overall survival (OS) as a function of constructed biologic subtypes from the diagnosis of ILRR.

The actuarial 5-year LRC rate was 77.3% for the overall cohort.  Table 4 lists the 5-year LRCs as stratified by patient characteristics and treatments. A Kaplan-Meier analysis of LRC as stratified by constructed breast cancer biologic subtype is showed in Figure 1(a). The actuarial 5-year LRC was 84.2% for Rec+/HER2-, 80.4% for Rec+/HER2+, 74.1% for Rec-/HER2+, and 58.3% for Rec-/HER2- patients (P < 0.001), respectively.

Moreover, 165 patients (61.3%) subsequently developed distant metastatic disease. Among these, 114 patients had DM alone, 51 patients had DM with concomitant LRF. Only 5 patients were identified to have isolated LRF. Bone, liver, lung, and brain were the most common sites of distant dissemination.

### 3.2. Survival Outcomes

At the end of follow-up period, 126 patients had died and the others were alive. The median OS from primary diagnosis for all patients was 100.2 (33.1-281.4) months; the actuarial 5- and 10-year survival rates after primary treatment were 89.5% and 54.8%, respectively. The median OS after ILRR was 64.8 (19.5-143.1) months, and the 5-year OS rate was 66.8%. The median DMFS after ILRR was 47.1 (5.2-143.1) months, and the 5-year DMFS was 45.6%. The median DFS after ILRR was 44.5 (5.2-143.1) months, and the 5-year DFS rate was 43.9%.

Figures 1(b)–1(d) presented Kaplan-Meier analyses of DMFS, DFS, and OS as stratified by constructed breast cancer biologic subtype. The 5-year DMFS was 63.0% for Rec+/HER2-, 32.5% for Rec+/HER2+, 16.7% for Rec-/HER2+, and 15.8% for Rec-/HER2- patients (P < 0.001); and the DFS at 5 years was 59.7% for Rec+/HER2-, 31.7% for Rec+/HER2+, 16.7% for Rec-/HER2+, and 13.6% for Rec-/HER2- patients (P < 0.001), respectively. An analysis of 5-year OS demonstrated similar trends between constructed biologic subtypes with rates of 87.8% for Rec+/HER2-, 67.5% for Rec+/HER2+, 36.5% for Rec-/HER2+, and 22.3% for Rec-/HER2- patients (P < 0.001).

### 3.3. Prognostic Factors Analysis

The correlation of 5-year LRC, DMFS, DFS, and OS with the various prognostic factors is listed in Table 4. On univariate analysis, multiple factors, including constructed biologic subtype, radical surgery, irradiated volume, and systemic treatment after ILRR, were significantly associated with LRC following RT for ILRR. Nodal status at primary diagnosis, constructed biologic subtype, disease-free interval, site of ILRR, radical surgery for ILRR, and systemic treatment for ILRR were significantly associated with both DMFS and DFS; in contrast, primary tumor stage, irradiated volume, and radiation dose were not associated with either DMFS or DFS. Similarly, nodal status at primary diagnosis, constructed biologic subtype, disease-free interval, site of ILRR, and systemic treatment for ILRR were significantly associated with OS. As summarized in Table 4, the constructed breast cancer biologic subtype was demonstrated to be the most significant factor affecting patient outcomes.

On multivariate analysis, constructed biologic subtype, radical surgery for ILRR, and irradiated volume remained to be statistically significant in predicting LRC (Table 5); Rec-/HER2-, no radical surgery for ILRR, and involved-field irradiation alone were significantly associated with reduced LRC. Nodal status at primary diagnosis, constructed biologic subtype, and site of ILRR remained to be statistically significant in the prediction of DMFS, DFS, and OS, while radical surgery for ILRR was of borderline significance in predicting DFS; positive nodes at primary diagnosis, non-Rec+/HER2-, and regional recurrence were significantly associated with poorer DMFS, DFS, and OS (Table 5). Obviously, non-Rec+/HER2- disease, particularly Rec-/HER2-, was the clinical parameter most strongly predictive for adverse outcomes, with hazard ratios (HR) of 4.36 (2.32-8.20) for LRC (P < 0.001), 4.28 (2.85-6.44) for DMFS (P < 0.001), 3.92 (2.64-5.84) for DFS (P < 0.001), and 8.55 (5.40-13.52) for OS (P < 0.001) at 5 years.

## 4. Discussion

The present analysis demonstrates that the most common pattern of subsequent failure is distant dissemination, rather than second LRR or locoregional progression, after salvage therapy for this cohort of 269 patients with postmastectomy ILRR. A number of independent prognostic factors, including primary characteristics (i.e., nodal status at primary diagnosis and constructed biologic subtype), recurrent characteristics (i.e., site of recurrence), and treatment-related factors (i.e., radical surgery, irradiated volume, and systemic therapy), were revealed to predict for clinical outcomes. Generally, the outcomes were poor in patients carrying unfavorable prognostic factors, such as positive nodes at primary diagnosis, Rec-/HER2-, and regional recurrence, but might be improved by the use of radical surgery and comprehensive locoregional irradiation

Notably, in this study, the breast cancer biologic subtype constructed according to the status of hormonal receptors and HER2 was demonstrated to be significant for any clinical outcome following salvage treatment for ILRR after mastectomy in both univariate and multivariate models. First, constructed biologic subtype was a significant predictor for the incidence of LRF. Second, constructed biologic subtype represented a significant predictor for the incidence of subsequent distant dissemination; non-Rec+/HER2- patients had significantly worse 5-year DMFS, indicating that non-Rec+/HER2- breast cancer might be more aggressive and prone to metastasize. Third, the impact of constructed biologic subtype remained significant for postrecurrence DFS; it was not surprising that patients with non-Rec+/HER2- breast cancer had significantly worse 5-year DFS due to their tendency to fail locoregionally and/or metastasize distantly. Lastly but most significantly, constructed biologic subtype was demonstrated to predict for OS; patients with non-Rec+/HER2- breast cancer had significantly worse 5-year OS despite the use of comprehensive locoregional and systemic therapies, which might be attributed to its aggressive nature. It is apparent that constructed breast cancer biologic subtype remained to be predictive for both disease control and survivals in patients with ILRR after mastectomy alone. More specifically, Rec-/HER2- patients who developed postmastectomy ILRR were at high risk of LRF and distant dissemination and tended to have worse DFS and OS than Rec+/HER2- patients. The current analysis therefore suggests that tumor biology inferred by breast cancer subtypes represents the most significant prognostic factor despite the ensuing salvage treatment for postmastectomy ILRR.

Apart from constructed biologic subtype, nodal status which represents another characteristic of the primary tumor was also proved to carry prognostic significance in this cohort of patients developing ILRR after mastectomy. Specifically, postmastectomy patients with positive nodes at their primary diagnosis sustained lower rates of LRF but had worse survivals than node-negative patients after multimodality therapy for ILRR. This finding is in line with previous reports, which demonstrated that postmastectomy patients with either higher tumor or nodal stages at primary diagnosis had a poor prognosis after ILRR than patients without these risks [3, 6] and thus provide further verification of their adverse effect on local disease control and survival outcomes.

The anatomical subsite of recurrence which represents one major characteristic of recurrent tumors was demonstrated to be an independent prognostic factor as well. In our study, patients with regional recurrence maintained a moderate LRC but had worse survivals than those with recurrence at CW alone. These findings are consistent with previous studies reporting that patients with CW recurrence alone did better than those with SCV/ICV, IMN recurrence, or recurrence at multiple regions [7, 14, 15]. Unfortunately, the current staging system has no mechanism to categorize patients with postmastectomy ILRR. In would be of great interest to identify subgroups of mastectomized patients with ILRR differing in their prognosis based on classical clinic-pathological characteristics at the time of both primary and recurrent diagnosis, as well as on some new biological parameters.

The locoregional treatment strategies for patients with postmastectomy ILRR have been shown to significantly affect the postrecurrence outcomes. Our observations are similar to or even better than the published series on ILRR [3, 6, 16, 17], highlighting that aggressive comprehensive locoregional therapies are appropriate. Generally, patients with CW or axillary recurrence are often encouraged to be treated with comprehensive locoregional treatment, consisting of pre-RT surgery and locoregional irradiation, whereas most patients with IMN, SCV/ICV, or multiple site recurrence are often recommended to receive locoregional irradiation alone. As local management techniques have improved, treatment failures may have become increasingly attributable to aggressive tumor biology rather than an inability to identify and manage the residual disease. As such, overall recurrence rates would be expected to decline, while the nature of any recurrences would become increasingly refractory to treatment.

By contrast, the significance of using systemic therapy, especially chemotherapy, in the setting of postmastectomy ILRR has been controversial [6, 7]. In the present cohort of patients, systemic therapy was not found to be a significant predictor for either LRC or survivals, which might be ascribed to the limited number of patients without systemic therapy and existing disparities in patient subgroups and treatment details. However, the recently published data from the CALOR trial [16], which randomly assigned 162 patients who underwent surgical resection for locally recurrent breast cancer to postexcision chemotherapy versus no chemotherapy, suggested that there is a significant benefit from postexcision chemotherapy, particularly among patients with ER-negative disease. In addition, numerous efforts are underway to both reduce the risk of LRR and to treat recurrences [18]. Among these are trials that incorporate new hormonal or targeted agents to overcome resistance and concurrent radiosensitizing agents with adjuvant or salvage RT. With the accumulation of data on various systemic therapies for patients with different prognosis, it would be possible to select group of patients' candidates for individualized therapeutic strategies.

## 5. Conclusions

This cohort of breast cancer patients with postmastectomy ILRR achieved a long-term LRC and modest survivals after salvage RT +/- pre-RT surgery and systemic therapy. Constructed biologic subtypes remained to be predictive of both postrecurrence disease control and survivals. Notably, Rec-/HER2- patients were demonstrated to be at high risk of LRF and subsequent distant dissemination and tended to have worse DFS and OS despite ensuing salvage therapies. Clearly, more investigation is needed to improve the prognosis of patients with unfavorable prognostic factors.

## Figures and Tables

**Table 1:** Patient characteristics at time of primary diagnosis and initial treatment (n=269).

Parameters		Value
Median age (range) (years)		47 (26-83)
Menopausal status		
	Pre-/peri	162 (60.2%)
	Post-	107 (39.8%)
Location of primary tumor		
	Medial	90 (33.5%)
	Central	21 (7.8%)
	Outer	158 (58.7%)
Primary tumor histopathology		
	IDC	254 (94.4%)
	Others	15 (5.6%)
Primary tumor stage		
	pT1	87 (32.3%)
	pT2	157 (58.4%)
	pT3	25 (9.3%)
No. of nodes removed		13 (5-31)
Nodal stages		
	pN0	144 (53.5%)
	pN1	125 (46.5%)
Biologic subtype		
	Rec+/HER2-	158 (58.7%)
	Rec+/HER2+	26 (9.7%)
	Rec-/HER2+	24 (8.9%)
	Rec-/HER2-	61 (22.7%)
Primary surgery		
	MRM	228 (84.8%)
	Others	41 (15.2%)
		
Initial systemic therapy		
	Chemotherapy only	104 (38.7%)
	Endocrine therapy only	11 (4.1%)
	Chemotherapy + endocrine therapy	147 (54.6%)
	Anti-HER2	4 (1.5%)
	None	7 (2.6%)

IDC = invasive ductal carcinoma; Rec = hormonal receptor; HER2 = human epidermal growth factor receptor 2; MRM = modified radical mastectomy.

**Table 2:** Patient characteristics at time of recurrence and salvage treatment (n=269).

Parameters		Value
Median age (range) (years)		52 (27-85)
Interval to ILRR from initial diagnosis (mos)		33.5 (4.6-236.0)
Follow-up from the date of recurrence (mos)		64.7 (19.5-143.1)
Follow-up from the date of RT completion (mos)		59.1 (15.9-135.2)
Menopausal status		
	Pre-/peri-	104 (38.7%)
	Post-	165 (61.3%)
Site of ILRR		
	CW alone	90 (33.5%)
	Regional nodes alone	141 (52.4%)
	Axilla alone	15
	IMN alone	26
	SCV/ICV alone	77
	Multiple regions	23
	CW + regional nodes	38 (14.1%)
Multiple sites of recurrence		
	Yes	61 (22.7%)
	No	208 (77.3%)
Locoregional treatment for recurrence		
	RT alone	189 (70.3%)
	Palliative surgery + RT	20 (7.4%)
	Radical surgery + RT	60 (22.3%)
Irradiated volume		
	CW alone	12 (4.5%)
	Regional nodes alone	16 (5.9%)
	CW and regional nodes	241 (89.6%)
	SCV/ICV	156 (58.0%)
	SCV/ICV+IMN	68 (25.3%)
	SCV/ICV+Axilla	14 (5.2%)
	SCV/ICV+IMN+Axilla	3 (1.1%)
Radiation dose (Gy)		60 (46-66)
	<50	38 (14.1%)
	50-60	182 (67.7%)
	>60	49 (18.2%)
Systemic therapy after recurrence		
	None	13 (4.8%)
	Chemotherapy only	72 (26.8%)
	Endocrine therapy only	54 (20.1%)
	Chemotherapy + endocrine therapy	130 (48.3%)
	Anti-HER2	24 (8.9%)

ILRR = isolated locoregional recurrence; RT = radiation therapy; IMN = internal mammary nodes; CW = chest wall; SCV = supraclavicular; ICV = infraclavicular; others as in Table 1.

**Table 3:** Patterns of locoregional failure (LRF) after salvage radiotherapy for ILRR.

Parameters	Value
No. of patients	56
Incidence of LRF (%)	20.8%
Site of LRF	
CW alone (n)	28 (50.0%)
Regional nodes alone (n)	20 (35.7%)
CW and regional nodes (n)	8 (14.3%)
SCV/ICV	14
IMN	12
Axilla	1
Multiple regions	1
Association of locoregional failure and radiation field	
In filed	23 (41.1%)
Out of field	20 (35.7%)
Both	13 (23.2%)

LRF = locoregional failure. Others as in Table 2.

**Table 4:** Results of univariate analysis of patient characteristics and treatment-related factors for outcomes of patients with ILRR.

Parameters	5-year LRC after RT	P-value	5-year DMFS after ILRR	P-value	5-year DFS after ILRR	P-value	5-year OS after ILRR	P-value
Primary tumor stage								
T1-2	79.1%	0.120	46.7%	0.420	45.0%	0.450	68.4%	0.120
T3	64.2%		33.6%		31.1%		50.2%	
Nodal stage								
N0	77.8%	0.450	51.9%	**0.019**	49.6%	**0.020**	71.5%	**0.037**
N1	76.3%		38.0%		37.1%		60.4%	
Biologic subtype								
Rec+/HER2-	84.2%	**0.001**	63.0%	**0.001**	59.7%	**0.001**	87.8%	**0.001**
Rec+HER2+	80.4%		32.5%		31.7%		67.5%	
Rec-/HER2+	74.1%		16.7%		16.7%		36.5%	
Rec-/HER2-	58.3%		15.8%		13.6%		22.3%	
Disease-free interval (years)								
⩽2	78.3%	0.700	37.8%	**0.025**	36.9%	**0.048**	52.1%	**0.001**
>2	79.5%		50.3%		47.9%		75.9%	
Site of ILRR								
CW alone	79.4%	0.560	59.2%	**0.001**	56.9%	**0.002**	78.9%	**0.001**
Regional nodes +/- CW	70.2%		35.7%		36.1%		54.5%	
Radical surgery for ILRR								
Yes	86.1%	**0.04**	41.9%	**0.005**	56.5%	**0.005**	76.2%	0.100
No	74.4%		57.9%		39.9%		63.9%	
Irradiated volume								
CW or regional nodes alone	51.5%	**0.001**	40.5%	0.522	32.9%	0.170	61.8%	0.520
CW + regional nodes	80.7%		45.9%		45.1%		67.2%	
Radiation dose (Gy)								
⩽60	78.8%	0.640	45.9%	0.575	44.1%	0.520	69.8%	0.270
> 60	74.3%		43.0%		42.2%		53.3%	
Systemic treatment after ILRR								
Yes	79.4%	**0.040**	46.8%	**0.010**	45.0%	**0.009**	65.2%	**0.001**
No	67.3%		23.1%		23.2%		35.4%	

LRC = locoregional control; DMFS = distant metastasis-free survival; DFS = disease-free survival; OS = overall survival. Others as in Table 2.

**Table 5:** Multivariate analysis of prognostic factors for outcomes after postmastectomy ILRR.

Parameter	LRC			DMFS			DFS			OS		
	HR	95% CI	P-value	HR	95% CI	P-value	HR	95% CI	P-value	HR	95% CI	P-value
Nodal stage												
Node-positive versus node-negative				1.65	1.19-2.27	**0.002**	1.62	1.18-2.23	**0.003**	1.72	1.19-2.48	**0.001**
Biologic subtype			**0.001**			**0.001**			**0.001**			**0.001**
Rec+/HER2-												
Rec+/HER2+	1.48	0.56-3.88	0.424	2.28	1.31-3.98	0.004	2.10	1.23-3.61	0.009	3.09	1.68-5.71	0.001
Rec-/HER2+	1.95	0.73-5.18	0.181	3.91	2.33-6.58	0.001	3.55	2.16-5.83	0.001	5.77	3.25-10.22	0.001
Rec-/HER2-	4.36	2.32-8.20	0.001	4.28	2.85-6.44	0.001	3.92	2.64-5.84	0.001	8.55	5.40-13.52	0.001
Disease-free interval (years)												
≤ 2 versus >2				0.95	0.68-1.32	0.773	0.97	0.70-1.36	0.891	0.84	0.58-1.23	0.387
Regional recurrence												
Yes vs. no				2.03	1.06-3.41	**0.033**	1.59	1.04-2.69	**0.045**	1.41	1.02-2.50	**0.052**
Radical surgery for ILRR												
Yes versus No	0.37	0.18-0.82	**0.009**	0.67	0.34-1.32	0.256	0.64	0.41-1.02	**0.063**	0.74	0.44-1.24	0.253
Irradiated volume												
CW or regional nodes												
CW + regional nodes	0.26	0.14-0.49	**0.001**									
Systemic treatment after ILRR												
Yes versus No	0.63	0.21-1.84	0.402	0.67	0.34-1.32	0.255	0.64	0.32-1.27	0.202	0.56	0.29-1.09	0.082

HR = hazard ratio. Others as in Table 4.

## Data Availability

The data used to support the findings of this study are available from the corresponding author upon request.
